# The First Impact Factor - *Journal of Intensive Care*

**DOI:** 10.1186/s40560-020-00467-y

**Published:** 2020-07-17

**Authors:** Hiroshi Morisaki

**Affiliations:** Department of Anesthesiology, Keio University School of Medicine, 35 Shinanomachi, Shinjuku-ku, Tokyo, 160-8582 Japan

As Editor-in-Chief, I am pleased to inform you that the *Journal of Intensive Care* (JINC) has been granted its first official impact factor of “3.103” in the 2019 Journal Citation Reports lately released by Clarivate Analytics. In the reports, the JINC is ranked 14^th^ out of 36 journals in the category of Critical Care Medicine. With this joyous announcement, I would like to congratulate all of you who were, are, or will be involved with our journal.

The *Journal of Intensive Care* (JINC), launched in October 2013 as the official journal of the Japanese Society of Intensive Care Medicine (JSICM), is an open access journal encompassing all aspects of intensive care medicine, such as intensive and critical care, trauma and surgical intensive care, and pediatric intensive care. In addition, the journal encourages submissions considering the different cultural aspects of intensive care practice. Through the close collaboration of the JSICM with the Society of Critical Care Medicine and the European Society of Intensive Care Medicine, the JINC has aimed to function as a leading international journal in the field of Intensive Care, alongside *Critical Care Medicine* and *Intensive Care Medicine*, both of which are official journals of the respective societies. Thus far, we have persistently served the community through the timely sharing of important and valuable research findings that arouse discussion and promote further research activities. We have received considerable contributions by numerous authors and/or researchers who submitted their manuscripts to our journal. Consequently, we could have published a variety of inspiring articles that have provided substantial influence on the field of intensive care and related areas.

Last year, our acceptance rate for research and review articles were 16.2% and 20.8%, respectively. Together with the other article types such as case reports which hardly gets published in this journal, the overall acceptance rate in 2019 was 15.6%, showing a deep decrease from 21.3% in 2017 [1]. Furthermore, the average intervals from the formal receipt to the dates of the first and final decisions were 13.1 and 24.4 days, respectively. Regardless of the state of the editorial process, I believe that we have returned high-quality reviewers’ comments to authors through dedicated and suitable contributions by peer reviewers. To this end, I express our sincere appreciation to all of our peer reviewers.

However, receiving an impact factor is not our goal but rather a stepping-stone in our drive to become a valuable, influential international journal in this field. We look forward to continuing to publish high-quality research and review articles that will contribute to the significant improvement of the field of intensive care.
